# Reprogramming of Treg cells in the inflammatory microenvironment during immunotherapy: a literature review

**DOI:** 10.3389/fimmu.2023.1268188

**Published:** 2023-09-11

**Authors:** Xinyan Wu, Zhigang Zhou, Qiang Cao, Yuquan Chen, Junling Gong, Qi Zhang, Yi Qiang, Yanfeng Lu, Guangzhu Cao

**Affiliations:** ^1^ Department of Earth Sciences, Kunming University of Science and Technology, Kunming, China; ^2^ College of Veterinary Medicine, Sichuan Agricultural University, Chengdu, China; ^3^ Department of Oncology, Changde Hospital, Xiangya School of Medicine, Central South University, Changde, China; ^4^ School of Medicine, Macau University of Science and Technology, Macau, Macau SAR, China; ^5^ Institute of Medical Information/Library, Chinese Academy of Medical Sciences, Beijing, China; ^6^ School of Public Health, Nanchang University, Qianhu, Nanchang, China; ^7^ Undergraduate Department, Taishan University, Taian, China

**Keywords:** inflammatory microenvironment, Tregs, Foxp3, immunotherapy, cancer progression, immune evasion

## Abstract

Regulatory T cells (Treg), as members of CD4+ T cells, have garnered extensive attention in the research of tumor progression. Treg cells have the function of inhibiting the immune effector cells, preventing tissue damage, and suppressing inflammation. Under the stimulation of the tumor inflammatory microenvironment (IM), the reprogramming of Treg cells enhances their suppression of immune responses, ultimately promoting tumor immune escape or tumor progression. Reducing the number of Treg cells in the IM or lowering the activity of Treg cells while preventing their reprogramming, can help promote the body’s anti-tumor immune responses. This review introduces a reprogramming mechanism of Treg cells in the IM; and discusses the regulation of Treg cells on tumor progression. The control of Treg cells and the response to Treg inflammatory reprogramming in tumor immunotherapy are analyzed and countermeasures are proposed. This work will provide a foundation for downregulating the immunosuppressive role of Treg in the inflammatory environment in future tumor immunotherapy.

## 1 Introduction

With the successful implementation of immune checkpoint inhibitors, such as those targeting cytotoxic T-lymphocyte-associated protein 4 (CTLA-4) and programmed death receptor 1 (PD-1), immunotherapy has become a pivotal cornerstone in the realm of cancer treatment (1–3). The immune system consists of various immune cells, among which T cells are the main cell types exerting anti-tumor effects during the adaptive immune phase (4–6). T cells are divided into CD4+ T cells (also known as helper T cells) and CD8+ T cells, primarily located in lymph nodes and serving patrol and surveillance functions. Once activated by antigen-presenting cells, CD4+ T cells can quickly differentiate into different subtypes, among which those with immunosuppressive and pro-tumoral functions are referred to as regulatory T cells(Treg) (7–9). CD4+ T cells can differentiate into various subtypes, including Th1 and Th2 cells, known for secreting interferon-γ and interleukin, respectively.CD4+ T cells that can secrete interleukin-17 are referred to as TH17. Activated immune cells and tumor cells share similar metabolic pathways and can undergo a phenomenon termed “metabolic reprogramming” (10–12). The tumor microenvironment (TME) shows varying degrees and types of immune cell infiltration. The high metabolism of tumor cells and the disordered vascular system within the TME lead to nutrient exhaustion and hypoxic conditions, setting up metabolic competition between tumor cells and infiltrating immune cells (13–15). The activation process of immune cells requires a substantial amount of energy and metabolic intermediates to meet the needs of biosynthesis, thereby completing proliferation, differentiation, and execution of effector functions (12, 16). The nutrient exhaustion caused by tumor cells and immune cells competing for the same energy sources, along with the inflammatory microenvironment in the TME promoting Treg cell reprogramming, ultimately leads to immune escape and tumor progression (17, 18).

Inflammation has long been a significant factor in the occurrence and development of cancer. Hanus’s study (19) suggests that cancer incidence is closely related to the levels of inflammatory cytokines and the compositional structure of immune cells. The relationship between cancer incidence and immune cell composition is based on studies suggesting that the type and abundance of immune cells within the tumor microenvironment can influence various aspects of cancer development, including cell proliferation, angiogenesis, and metastasis (20, 21).. In inflamed tissues, immune cells and cytokines have a regulatory role in preventing excessive tissue damage (22–24). When immune cell function is abnormal, it may promote “inflammation-to-cancer transformation.” Zhu’s study (25) elucidates potential interactions among immune cells in the tumor microenvironment during cancer development and progression, including changes in cell ratios, crosstalk, and changes in the plasticity of immune cell phenotypes. Among these immune cells, the reprogramming of Tregs is an important regulatory factor in immune responses and inflammatory diseases. Naive CD4+ T cells bind to the major histocompatibility complex II (MHC II) expressed by innate immune cells, regulating helper T cell (Th) differentiation via co-stimulatory molecules and the release of inflammatory cytokines (26–28). Typically, Tregs undergo reprogramming under the induction of the inflammatory environment and suppress anti-tumor immune responses (29, 30). This article will delve into the reprogramming of Tregs in an inflammatory environment and their role in tumor occurrence and development. Additionally, it will analyze how to avoid the impact of Treg reprogramming under inflammatory conditions in immunotherapy, based on this mechanism.

## 2 Location and physiological regulation of Treg cells

Tregs primarily comprise two groups: thymus-derived natural Tregs (nTregs), which originate in the thymus during T cell development, and peripherally induced Tregs (pTregs), which are generated in peripheral tissues. Tregs suppress the function of effector T cells via multiple pathways, including the production of immunosuppressive cytokines, such as TGF-β and IL-10. Through interactions with effector T cells or antigen-presenting cells (APCs) and other immune cells, they play a crucial role in establishing and maintaining immune tolerance. nTregs typically appear in peripheral blood and can mitigate graft-versus-host disease (GVHD) and autoimmune diseases (31–33).

**Figure 1:**
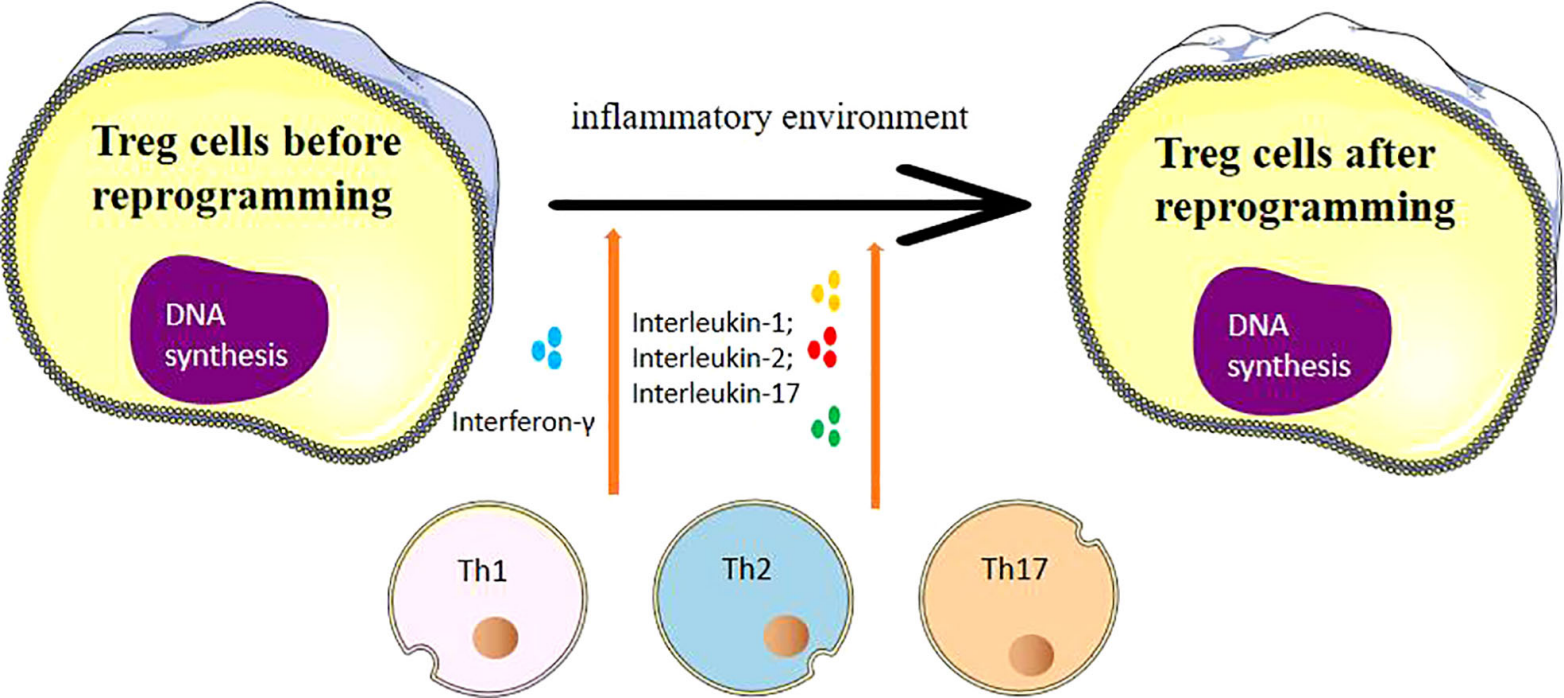
The process of Treg cell reprogramming and the influencing factors suffered.

Immune cells Th17 and Treg both originate from naive CD4+ T cells and can be induced to differentiate by TGF-β. However, they express different gene transcription factors and have distinct effector functions. Tregs and Th17 are generally considered to play opposing roles in immune regulation, with key transcription factors being the decisive elements in guiding the differentiation of CD4+ T cells into Tregs and Th17 (34, 35). Mickael etal’s study (36) indicates that the transcription factor Foxp3 is pivotal in Tregs exerting immunosuppressive functions. It can inhibit the expression of Th17’s transcription factors, retinoic acid receptor-related orphan receptor γt (RORγt), and RORα, thus promoting Treg differentiation. As shown in **Figure 1**, several pro-inflammatory factors, including IL-1, IL-2, and IL-17, have been associated with promoting Th1, Th2 and Th17 differentiation, under certain conditions, can influence Treg reprogramming and affect the expression of FOXP3. Tregs residing in tissues, such as tumor microenvironments, can exert intricate regulatory effects that differ from their counterparts circulating in peripheral blood. Tissue-resident Tregs are poised to modulate localized immune responses, impacting tumor progression and inflammation, whereas circulating Tregs may play broader systemic roles. The tumor microenvironment exerts a multifaceted influence on Treg reprogramming through a combination of cytokine signaling, metabolic competition, exosome-mediated communication, hypoxia-induced stabilization, and interactions with other immune cells. Understanding these dynamic interactions provides a deeper insight into how Tregs adapt to and modulate the TME, ultimately contributing to tumor immune escape and progression. In the differentiation process of Tregs and Th17, the discovery of new subgroups also provides important insights for disease exploration (37–39). For instance, IL-17+FOXP3+ T cells produced in chronic inflammatory environments can express FOXP3 and secrete IL-17 simultaneously. Multiple clinical studies have found that in autoimmune diseases, allergies, tumors, and other diseases, the reprogramming of Tregs under an inflammatory microenvironment significantly impacts disease progression and prognosis (40–42).

## 3 Mechanism of Treg reprogramming under inflammatory microenvironment

FOXP3 is a Treg cell-specific transcription factor that plays a critical regulatory role in the development and function of Tregs. The immune regulatory function of Tregs mediated by FOXP3 is achieved through the dynamic control of gene transcription by forming protein complexes with several co-regulatory transcription proteins. In CD4+ T cells, the co-expression of FOXP3 and a series of its associated proteins enables the cells to acquire a Treg phenotype (43–45). However, the independent expression of any single protein does not induce Treg-like gene expression, thereby demonstrating the significance of the collaborative efforts of FOXP3 and its co-regulatory molecules in cellular phenotype and function. FOXP3 can also form different complexes with different transcription factors, thereby affecting the specificity of Treg cell suppression function. In the IM, FOXP3 in Treg cells can combine with T-bet, a key transcription factor in Th1 cells, due to external environmental stimuli, specifically enhancing the immunosuppressive function of Tregs, which in turn promotes tumor progression (46–48).

The balance between lysine acetylation and deacetylation of the FOXP3 protein in Treg cells can dynamically regulate its immunosuppressive function. Under IM, histone acetyltransferases (HATs) TIP60 and p300 can bind and acetylate the FOXP3 protein. The acetylation-modified FOXP3 not only stabilizes the FOXP3 protein but also enhances its transcriptional activity and function, positively regulating the immunosuppressive activity of Treg cells (49–51). Consequently, Tregs acquire enhanced immunosuppressive activity after reprogramming, promoting tumor immune evasion or tumor progression (52, 53).

## 4 Regulation of tumor development by Treg infiltration into cancer tissues under inflammatory microenvironment

A study by Parajuli (54) indicates that an increase in Tregs facilitates immune evasion by the tumor. Introducing T cells without Tregs significantly improves the body’s anti-tumor immune response. Simultaneously, the proliferation or activation of FOXP3+ Tregs under IM severely inhibits tumor immunity. Clinically, the increase of Treg cells in tumor microenvironments such as lung adenocarcinoma, pancreatic cancer, and lymphoma correlates with inflammatory reprogramming and poor prognosis (55, 56). Mechanistically, Treg cells not only have the ability to suppress a broad range of anti-tumor immune responses, but also promote angiogenesis in the tumor microenvironment.

A large body of literature indicates a correlation between Treg accumulation in tumor tissues and poor prognosis, but some reports associate Tregs with better prognosis in diseases such as hepatocellular carcinoma and colon cancer (57). The functional heterogeneity of tumor-infiltrating Tregs, the site of infiltration, and detection methods, as well as factors such as levels of CD8+ cytotoxic T cells, tumor cell immunogenicity, and inflammatory infiltration of the tumor microenvironment, can explain these seemingly contradictory results. Sakowska etal’s study (58) points out that a class of prostaglandin E2 (PGE2)-secreting pTregs can proliferate extensively under the stimulation of tumor antigens and secrete inhibitory cytokines to suppress anti-tumor immune responses. At the same time, in tumors with inflammatory infiltration, this type of iTreg can downregulate inflammatory responses, thereby preventing tissue damage and tumor development. In a study of patients with lung adenocarcinoma, it was found that the number of Tregs infiltrating the para-cancerous tissues was positively correlated with tumor development, while patients with Tregs infiltrating the cancerous tissues had a better prognosis (59, 60).

## 5 Addressing Treg proliferation and inflammatory reprogramming in tumor immunotherapy

Under IM, tumor-infiltrating Tregs and their inflammatory reprogramming in the inflammatory milieu contribute to tumor immune evasion, making Tregs an important target in tumor immunotherapy. The currently employed methods are primarily focused on eliminating Tregs; blocking chemokines or their receptors to prevent the migration of Tregs towards the inflammatory tumor microenvironment; abolishing the IM to inhibit the induction of Tregs’ inflammatory reprogramming; obstructing key surface markers of Tregs such as immune checkpoints to reduce the suppressive function of Tregs (61–64).

### 5.1 Reducing the immunosuppressive function of Tregs

As shown in **Figure 1**, Cytotoxic T-Lymphocyte Associated Protein 4 (CTLA-4) is a marker expressed on the surface of activated T cells that transmits inhibitory signals during immune responses. CTLA-4 is constitutively expressed on the surface of Tregs, and its expression is upregulated after TCR stimulation. Some study has proven that CTLA-4 can reduce the immunosuppressive activity of Tregs. The humanized anti-CTLA-4 monoclonal antibody, Ipilimumab (Yervoy), is currently used to treat advanced metastatic melanoma.Furthermore, it’s important to note that CTLA-4, while expressed on Tregs, also has implications for conventional T cells. CTLA-4 engagement suppresses the activation and effector functions of conventional T cells, thereby contributing to overall immune regulation (65, 66).

OX40, a costimulatory molecule of the TNF receptor family, is transiently expressed on the surface of activated T cells and constitutively expressed on the surface of Treg cells. Activating the OX40 signaling pathway with anti-OX40 monoclonal antibodies in an inflammatory microenvironment can reduce the immunosuppressive activity of Tregs, thereby reducing their immunosuppressive function (67, 68).

Programmed death receptor 1 (PD-1) negatively regulates the activation status of T cells. PD-1 promotes the development of Tregs and is mainly highly expressed on the surface of T cells that cannot effectively participate in the anti-tumor immune response. Although the main purpose of PD-1 ligand blockade is to reverse the exhaustion state of T cells, PD-1 ligand blockade can also hinder the development of Tregs and prevent Treg reprogramming under inflammatory conditions. So far, the anti-PD-1 monoclonal antibody Pembrolizumab has been used to treat lung cancer, gastric cancer, and cervical cancer (69, 70).

GITR, a member of the TNF receptor family, is expressed at low levels in CD4+FOXP3- T cells and constitutively at high levels in Treg cells. When Tregs infiltrate tumors, the expression level of GITR is even higher. GITR ligands can specifically reduce Tregs in the tumor, increase the ratio of effector T cells to Tregs, and thereby decrease the immunosuppressive function of Tregs in the inflammatory microenvironment, improving the effects of immunotherapy (18, 71).

### 5.2 Treg depletion

Treg depletion therapy has shown vast potential. This treatment strategy primarily targets the IL-2 receptor chain, and the anti-CD25 monoclonal antibody can block the IL-2 signaling pathway by binding to CD25, thereby causing Treg cell death. Recombinant immunotoxins (RITs), such as scFv-psm-ETA, can also cause massive Treg depletion, thus affecting Treg function. However, using RITs to deplete Tregs has strong side effects, as this treatment method affects CD4+CD25hi effector T cells, further weakening the body’s anti-tumor immunity and increasing the risk of the patient developing autoimmune diseases (72, 73).

In addition to depleting Tregs by targeting Treg-specific surface markers, some chemotherapy drugs can also achieve Treg depletion by reducing Treg proliferation. These drugs include antimicrotubule agents, such as cyclophosphamide, docetaxel, vincristine, and thalidomide analogs, as well as cyclooxygenase-2 (COX2) inhibitors. Cyclophosphamide can alkylate DNA, causing DNA cross-linking and cell death. Reports have stated that Treg cells are highly sensitive to cyclophosphamide-induced apoptosis.Regarding T cell receptors (TCRs) of Tregs within tumor tissues, an intriguing area of investigation emerges. Tregs play a pivotal role in maintaining immune homeostasis and preventing autoimmunity, and their presence in tumor microenvironments raises questions about their TCR diversity and specificity. TCRs on Tregs could potentially influence their interactions with tumor antigens, other immune cells, and the overall immunosuppressive milieu. Research into the TCR profiles of Tregs within tumor tissues could shed light on their potential dual role in dampening excessive immune responses while also contributing to tumor immune evasion. Exploring the TCR repertoire and antigen specificity of tumor-infiltrating Tregs may uncover new avenues for immunotherapy strategies and further enhance our understanding of the complex regulatory network in the tumor microenvironment. A study by Jiang (74) has shown that a single injection of cyclophosphamide can significantly reduce tumor growth rate. Both docetaxel and vincristine can inhibit DNA synthesis. A phase I clinical study showed that docetaxel can increase CTL in colorectal cancer patients while simultaneously reducing CD25+CD4+ T cells, thereby enhancing the body’s antiviral response. On the other hand, vincristine can inhibit the proliferation of IL-10 secreting Tregs ex vivo, while upregulating antigen-specific CTL (75, 76). Thalidomide and its derivatives can be used to treat multiple myeloma (77). Combinations of Treg-depleting agents with immune checkpoint inhibitors or other immunomodulatory therapies might create synergistic effects, amplifying the anti-tumor immune response and potentially reducing the doses of individual agents, thereby minimizing side effects.In addition to the strategies for modulating Tregs, emerging approaches like nanomedicine have shown promise in this realm (78–80). Nanomedicine offers innovative tools for precise targeting and modulation of immune cell populations, including Tregs, within the intricate tumor microenvironment. Leveraging these advancements, future immunotherapy research could explore novel ways to regulate Treg reprogramming and function, while minimizing off-target effects and maximizing therapeutic impact. As such, the integration of nanomedicine-based interventions to selectively counteract Treg reprogramming could open new avenues for enhancing the potency of cancer immunotherapy strategies. A clinical trial for chronic leukemia showed that a thalidomide derivative, lenalidomide, can significantly reduce the level of Tregs in peripheral blood and increase the number of Th17 cells. It is known that COX2 can affect tumor progression in many ways. Research indicates that COX-2 inhibitors can reduce the proportion and activity of Tregs in the IM, thereby achieving Treg depletion and enhancing the effects of immunotherapy (81–84).

## 6 Existing issues and prospects

Although research on immunotherapy and Tregs has made rapid progress in recent years, and the role and pathways of tumor-infiltrating Tregs in the tumor progression under the inflammatory microenvironment are continually being clarified, the current drugs used for removing or inhibiting the function of Treg cells, or for eliminating Tregs, generally have strong side effects or are less effective. There is still a need to continuously search for or develop new drugs that can suppress or eliminate Tregs, while having fewer side effects and better efficacy.

## 7 Conclusion

In the inflammatory microenvironment (IM), the binding of FOXP3 in Treg cells to T-bet leads to Treg reprogramming, which specifically enhances the suppressive function of Tregs on immune responses. In current cancer immunotherapy, Tregs have become important targets. To prevent tumor progression and immune evasion caused by Tregs, it is necessary to reduce the adverse effects of Treg reprogramming under inflammatory conditions on immunotherapy by reducing the immunosuppressive function of Tregs and eliminating Tregs. However, current methods either have strong adverse drug effects or are Less effective. Therefore, there is a need to continually develop drugs that can better clear or suppress Tregs with fewer side effects, thereby reducing the immunosuppressive effect of Tregs during the process of immunotherapy.

## Author contributions

XW: Conceptualization, Data curation, Formal Analysis, Funding acquisition, Investigation, Methodology, Project administration, Resources, Software, Supervision, Validation, Visualization, Writing – original draft, Writing – review & editing. ZZ: Conceptualization, Data curation, Formal Analysis, Funding acquisition, Investigation, Methodology, Project administration, Resources, Software, Supervision, Validation, Visualization, Writing – original draft, Writing – review & editing. QC: Conceptualization, Data curation, Formal Analysis, Funding acquisition, Investigation, Methodology, Project administration, Resources, Software, Supervision, Validation, Visualization, Writing – original draft, Writing – review & editing. YC: Conceptualization, Data curation, Formal Analysis, Funding acquisition, Investigation, Methodology, Project administration, Resources, Software, Supervision, Validation, Visualization, Writing – original draft, Writing – review & editing. JG: Conceptualization, Data curation, Formal Analysis, Funding acquisition, Investigation, Methodology, Project administration, Resources, Software, Supervision, Validation, Visualization, Writing – original draft, Writing – review & editing. QZ: Conceptualization, Data curation, Formal Analysis, Funding acquisition, Investigation, Methodology, Project administration, Resources, Software, Supervision, Validation, Visualization, Writing – original draft, Writing – review & editing. YQ: Conceptualization, Data curation, Formal Analysis, Funding acquisition, Investigation, Methodology, Project administration, Resources, Software, Supervision, Validation, Visualization, Writing – original draft, Writing – review & editing. YL: Writing – review & editing, Conceptualization, Data curation, Formal Analysis, Funding acquisition, Investigation, Methodology, Project administration, Resources, Software, Supervision, Validation, Visualization, Writing – original draft. GC: Conceptualization, Data curation, Formal Analysis, Funding acquisition, Investigation, Methodology, Project administration, Resources, Software, Supervision, Validation, Visualization, Writing – original draft, Writing – review & editing.
